# *catena*-Poly[oxidanium [tris{μ-[amino(iminio)methyl]phosphonato}zincate(II)]]

**DOI:** 10.1107/S2414314622002474

**Published:** 2022-03-10

**Authors:** Elpiniki Chachlaki, Duane Choquesillo-Lazarte, Konstantinos D. Demadis

**Affiliations:** a Crystal Engineering, Growth and Design Laboratory, Department of Chemistry, University of Crete, Voutes Campus, Crete, GR-71003, Greece; b Laboratorio de Estudios Cristalográficos, IACT, CSIC-Universidad de Granada, Granada-18100, Spain; Sunway University, Malaysia

**Keywords:** phospho­nate, coordination polymer, zinc, one-dimensional network, crystal structure

## Abstract

The crystal structure of the anionic zinc–[amino­(iminio)meth­yl]phospho­nate one-dimensional coordination polymer, Zn-AIMP, is reported; the negative charge is balanced by an oxidanium cation (H_3_O^+^) to give the composition {(H_3_O)[Zn(CH_4_N_2_PO_3_)_3_]}_*n*_. The building unit of the coordination polymer comprises a divalent Zn^2+^ cation (site symmetry  ..) and three [amino(iminio)meth­yl]phospho­nate mono-anionic ligands (point group symmetry *m*). The AIMP ligand exists in a zwitterionic form with a total charge −1 as the phospho­nate is fully deprotonated (–PO_3_^2−^), while the amino­(iminio)methyl moiety is protonated (H_2_N—C—NH_2_^+^).

## Abstract 2

The crystal structure of the anionic zinc–[amino­(iminio)meth­yl]phospho­nate one-dimensional coordination polymer, Zn-AIMP, is described.

## Graphical abstract

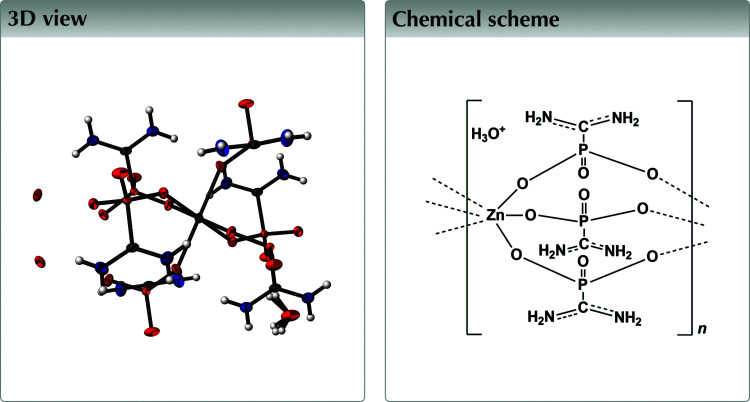

## Structure description

The chemistry of phospho­nic acids was initiated by the need for hydrolysis-resistant replacements for polyphosphates. Synthetic access to a variety of phospho­nic acid structures is possible through several well-established routes (Sevrain *et al.*, 2017 ▸). To the inorganic chemist, phospho­nic acids are a valuable synthetic tool as versatile ligands for generating a plethora of metal phospho­nate compounds that present diverse structural architectures, from molecular complexes, to chains and layers, to framework structures (Clearfield & Demadis, 2012 ▸). Herein, we report a new Zn^II^ phospho­nate one-dimensional anionic coordination polymer that contains the ligand [amino­(iminio)meth­yl]phospho­nate ({[Zn(CH_4_N_2_PO_3_)_3_]^−^}_*n*_, Zn-AIMP) and an oxidanium (H_3_O^+^) cation. The ligand AIMP was generated *in situ* during the synthesis by the decomposition of the hexa­ethyl 1,3,5-triazine-2,4,6-triyltris(phospho­nate) ester upon de­alkyl­ation with tri­methyl­bromo­silane.

The crystal structure of amino­(iminio)meth­yl]phospho­nate (obtained by decomposition of the ester hexa­ethyl 1,3,5-triazine-2,4,6-triyltris(phospho­nate) *via* acid hydrolysis and subsequent heating at 373 K) has been reported in the literature (Yang *et al.*, 2010 ▸). Inter­estingly, the sulfonate analogue of AIMP, amino­imino­methane­sulfonic acid (NH_2_)_2_CSO_3_ has been reported, and its crystal structure shows that this is also a zwitterion (Makarov *et al.*, 1999 ▸). Our de­alkyl­ation approach of the hexa­ethyl 1,3,5-triazine-2,4,6-triyltris(phospho­nate) ester to yield the acid under mild conditions and with the use of tri­methyl­bromo­silane did not lead to the desired (1,3,5-triazine-2,4,6-tri­yl)tris­(phospho­nic acid) product, but to AIMP.

**Figure 1:**
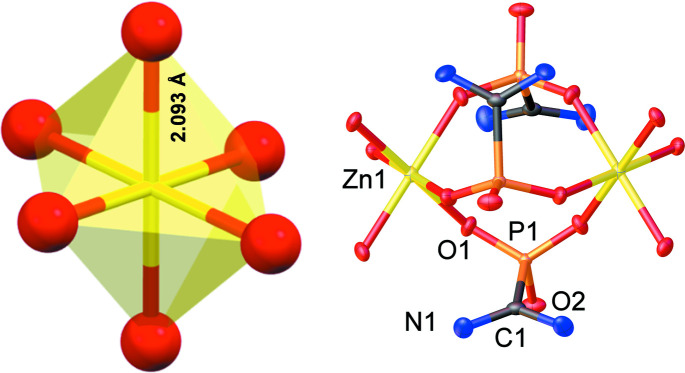
(left) The octa­hedral environment of the Zn^2+^ cation. (right) The bridging AIMP^−^ ligands with numbering scheme (H atoms and the disordered oxidanium cation are omitted for clarity). Displacement ellipsoids are shown at the 50% probability level. Colour codes: Zn yellow, P orange, O red, C black, N blue.

AIMP exists as a zwitterion in acidic solutions and it is neutral. However, at the pH of the reaction with Zn^II^, its second phospho­nic acid group is deprotonated, thus generating the AIMP anion. The Zn:AIMP molar ratio in Zn-AIMP is 1:3. Upon careful examination, the +2 charge of Zn^II^ is off-set by three mono-anionic AIMP ligands, offering a total charge of −3. In the absence of any other cations in solution, the excess −1 charge per building unit is balanced by an oxidanium cation that is generated by protonation of water (from the solvent). Zn-AIMP is a one-dimensional coordination polymer, its chains extending parallel to the *c* axis. The Zn^2+^ cation has a slightly distorted octa­hedral geometry, as illustrated in Fig. 1 ▸, coordinated exclusively by six phospho­nate oxygen atoms from six different AIMP ligands. The Zn—O distance is 2.0927 (16) Å, which falls in the expected Zn—O(phospho­nate) range (Colodrero *et al.*, 2010 ▸). Each AIMP ligand bridges two neighbouring Zn^2+^ cations, Fig. 1 ▸.

The phospho­nate group in the AIMP ligand is fully deprotonated, while the N—C—N moiety is protonated, hence each N atom bears two H atoms. From symmetry, the C1—N1 bonds are equivalent, with the bond length at 1.310 (3) Å being inter­mediate between those of a single and a double bond. The C—N bond length is comparable to that found in ‘free’ AIMP [1.299 (5) Å and 1.314 (5) Å; Yang *et al.*, 2010 ▸].

**Figure 2:**
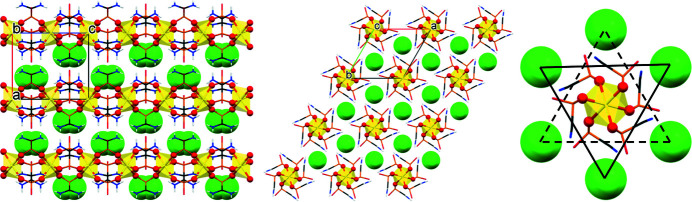
Packing of Zn-AIMP along the *b* axis (left) and along the *c* axis (middle). Arrangement of the H_3_O^+^ staggered triangles (right). The disordered H_3_O^+^ cations are shown as exaggerated green spheres. Colour coding is the same as in Fig. 1 ▸.

The P—O bond lengths are 1.4957 (15) Å (coordinating) and 1.527 (2) Å (non-coordinating). It is reasonable to assume that the −2 charge on the phospho­nate group is delocalized over all three O atoms. However, the P1—O2 bond (non-coordinating) is substanti­ally longer than the P1—O1 bond (coordinating) and this can be rationalized by the formation of hydrogen bonds between O2 with two two N—H moieties and the oxidanium cation (see below). The packing of the chains in Zn-AIMP along the *b*- and *c*-axis directions is shown in Fig. 2 ▸ (left and middle). The linear chains (intra-chain Zn—Zn—Zn angle = 180°) are packed parallel to the *c* axis. The oxidanium cation sits close to the non-coordinating P—O moiety of the chain and close to the N—C—N moiety of the neighbouring chain. The arrangement of the oxidanium cations (viewed down the *c* axis) is better described as staggered triangles that are ∼4.75 Å apart, see Fig. 2 ▸ (right).

**Figure 3:**
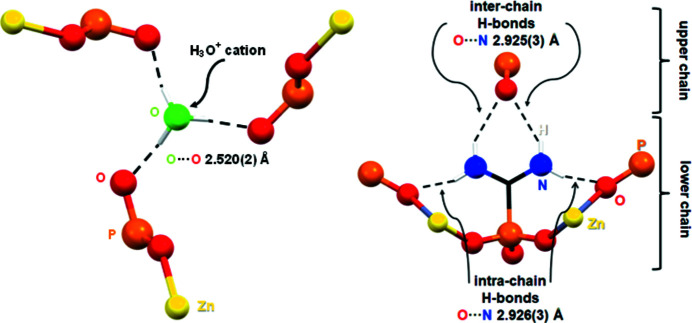
Hydrogen-bonding schemes in the structure of Zn-AIMP. (left) Hydrogen bonds between the disordered H_3_O^+^ cation and three non-coordinated O atoms from three different phospho­nate groups. (right) Intra-chain and inter-chain hydrogen bonds of the [H_2_N—C—NH_2_]^+^ moiety with phospho­nate O atoms.

The presence of several hydrogen-bond donors and acceptors in the structure creates hydrogen-bonding schemes that deserve some discussion, see Fig. 3 ▸. First, the H_3_O^+^ cation is located between the chains and utilizes all its H atoms to form three strong hydrogen bonds with three different non-coordinating phospho­nate O atoms originating from three neighbouring chains [O⋯O distance = 2.520 (3) Å, O3—H3⋯O2 angle = 155°, see Table 1 ▸ for symmetry codes]. Presumably, the H_3_O^+^ cations fill the intra-chain void space and stabilize the packing of the one-dimensional chains. It is noted the oxidanium-O3 atom, which is statistically disordered (see *Refinement*), does not form a close inter­action along the threefold axis it resides upon of less than 3.6 Å. In addition, the chains further inter­act *via* hydrogen bonds that include the cationic [H_2_N—C—NH_2_]^+^ moiety. Specifically, there are two intra-chain hydrogen bonds with Zn-coordinating phospho­nate O atoms [N⋯O distance = 2.926 (3) Å, N1—H1*B*—O2 angle = 147°] and two inter-chain hydrogen bonds with the non-coordinating phospho­nate oxygen from a neighboring chain [N⋯O distance = 2.925 (3) Å, N1—H1*A*—O1 angle = 143°].

## Synthesis and crystallization

**Reagents and materials** All starting materials were obtained from commercial sources and used without further purification. Ion-exchange column-deionized (DI) water was used for all syntheses. The starting reagents triethyl phosphite (98%), cyanuric chloride (98%) and zinc nitrate hexa­hydrate were from Alfa Aesar. The solvents petroleum ether, aceto­nitrile, methanol and nitric acid (70%) were from Scharlau. Tri­methyl­bromo­silane was from Flurochem.

**Syntheses of [amino­(iminio)meth­yl]phospho­nate (AIMP).** AIMP was synthesized from the de­alkyl­ation of the hexa­ethyl ester of 1,3,5-triazine-2,4,6-triyltris(phospho­nate). The latter was synthesized based on the synthetic procedure reported in the literature (Morrison, 1957 ▸) with modifications (Maxim *et al.*, 2010 ▸). Yield: 0.916 g, 92%. The ‘as synthesized’ solid ester (pure by NMR) was then de­alkyl­ated using tri­methyl­bromo­silane, as follows. In a dry vial the ester (0.490 g, 1.0 mmol) and tri­methyl­bromo­silane (1044 µ*L*, 8.0 mmol) were dissolved in aceto­nitrile (10 ml). The solution was stirred for 24 h, and the colour changed from faint orange to dark orange. Then the homogenous orange solution was left to stand at ambient temperature to allow evaporation of the solvent, yielding an orange oil. Methanol (10 ml) was added to remove the tri­methyl­silyl group from the phospho­nate moiety (as its meth­oxy ester), and the mixture was stirred for 1 h to allow precipitation of the desired AIMP product (Yield: 0.598 g, 60%). ^13^C NMR (75.5 MHz, DMSO-*d* ^6^) δ 169.71 (*d*). ^31^P NMR (121.5 MHz, DMSO-*d* ^6^) δ 2.85.

**Synthesis of {(H_3_O)[Zn(CH_4_N_2_PO_3_)_3_]}** ***_n_*** **(Zn-AIMP).** The synthesis of Zn-AIMP was performed at ambient temperature. Specifically, AIMP (0.016 g, 0.071 mmol); an excess was used, as it was found to give a product with better crystallinity) was dissolved in DI water (7 ml), Zn(NO_3_)_2_·6H_2_O (0.005 g, 0.017 mmol, dissolved in 1 ml DI water) was added, and the pH was adjusted to ∼3.5 using nitric acid. After 30 days a crystalline precipitate appeared, which was isolated by filtration and rinsed with a small amount of water (Yield: 0.001 g, 13%). The crystal used for measurement was handled under inert conditions, being manipulated while immersed in a perfluoro­polyether protecting oil, and was mounted on a MiTeGen Micromount™.

## Refinement

Crystal data, data collection and structure refinement details are summarized in Table 2 ▸. The oxygen atom of the H_3_O^+^ cation falls on a threefold axis and is disordered with respect to a mirror plane over two half-occupied O-atom positions. No further constraints were necessary to model the disorder.

## Supplementary Material

Crystal structure: contains datablock(s) I. DOI: 10.1107/S2414314622002474/tk4074sup1.cif

Structure factors: contains datablock(s) I. DOI: 10.1107/S2414314622002474/tk4074Isup2.hkl

Supporting information file. DOI: 10.1107/S2414314622002474/tk4074Isup3.mol

Supporting information file. DOI: 10.1107/S2414314622002474/tk4074Isup4.cdx

CCDC reference: 2151153

Additional supporting information:  crystallographic information; 3D view; checkCIF report

## Figures and Tables

**Table 1:** Hydrogen-bond geometry (Å, °)

*D*—H⋯*A*	*D*—H	H⋯*A*	*D*⋯*A*	*D*—H⋯*A*
N1—H1*A*⋯O2^i^	0.86	2.19	2.925 (3)	143
N1—H1*B*⋯O1^ii^	0.86	2.16	2.926 (3)	147
O3—H3⋯O2	0.98	1.59	2.520 (3)	155

Symmetry codes: (i) 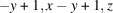 ; (ii) 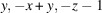 .

**Table 2:** Experimental details

Crystal data	
Chemical formula	(H_3_O)[Zn(CH_4_N_2_PO)_3_]
*M*_r_	453.49
Crystal system, space group	Hexagonal, *P*6_3_/*m*
Temperature (K)	298
*a*, *c* (Å)	9.5157 (15), 9.4946 (8)
*V* (Å^3^)	744.5 (2)
*Z*	2
Radiation type	Ag *K*α, λ = 0.56086 Å
μ (mm^−1^)	1.06
Crystal size (mm)	0.12 × 0.11 × 0.09

Data collection	
Diffractometer	Bruker D8 Venture
Absorption correction	Multi-scan (*SADABS*; Bruker, 2019 ▸)
*T*_min_, *T*_max_	0.684, 0.745
No. of measured, independent and observed [*I* > 2σ(*I*)] reflections	6814, 608, 534
*R*_int_	0.062
(sin θ/λ)_max_ (Å^−1^)	0.649

Refinement	
*R*[*F* ^2^ > 2σ(*F* ^2^)], *wR*(*F* ^2^), *S*	0.027, 0.072, 1.13
No. of reflections	608
No. of parameters	43
No. of restraints	1
H-atom treatment	H atoms treated by a mixture of independent and constrained refinement
Δρ_max_, Δρ_min_ (e Å^−3^)	0.28, −0.50

Computer programs: *APEX3* and *SAINT* (Bruker, 2019 ▸), *SHELXT* (Sheldrick, 2015*a* ▸), *SHELXL* (Sheldrick, 2015*b* ▸) and *OLEX2* (Dolomanov *et al.*, 2009 ▸).

## full crystallographic data

### Crystal data

**Table:**

(H_3_O)[Zn(CH_4_N_2_PO)_3_]	*D*_x_ = 2.023 Mg m^−^^3^
*M**_r_* = 453.49	Ag *K*α radiation, λ = 0.56086 Å
Hexagonal, *P*6_3_/*m*	Cell parameters from 1652 reflections
*a* = 9.5157 (15) Å	θ = 3.4–21.3°
*c* = 9.4946 (8) Å	µ = 1.06 mm^−^^1^
*V* = 744.5 (2) Å^3^	*T* = 298 K
*Z* = 2	Prism, colourless
*F*(000) = 460	0.12 × 0.11 × 0.09 mm

### Data collection

**Table:**

Bruker D8 Venture diffractometer	534 reflections with *I* > 2σ(*I*)
Radiation source: high brilliance microfocus sealed tube	*R*_int_ = 0.062
φ and ω scans	θ_max_ = 21.3°, θ_min_ = 2.6°
Absorption correction: multi-scan (SADABS; Bruker, 201)	*h* = −12→12
*T*_min_ = 0.684, *T*_max_ = 0.745	*k* = −12→10
6814 measured reflections	*l* = −12→12
608 independent reflections	

### Refinement

**Table:**

Refinement on *F*^2^	1 restraint
Least-squares matrix: full	Hydrogen site location: mixed
*R*[*F*^2^ > 2σ(*F*^2^)] = 0.027	H atoms treated by a mixture of independent and constrained refinement
*wR*(*F*^2^) = 0.072	*w* = 1/[σ^2^(*F*_o_^2^) + (0.0321*P*)^2^ + 0.6811*P*] where *P* = (*F*_o_^2^ + 2*F*_c_^2^)/3
*S* = 1.13	(Δ/σ)_max_ < 0.001
608 reflections	Δρ_max_ = 0.28 e Å^−^^3^
43 parameters	Δρ_min_ = −0.50 e Å^−^^3^

### Special details

**Table:**

Geometry. All esds (except the esd in the dihedral angle between two l.s. planes) are estimated using the full covariance matrix. The cell esds are taken into account individually in the estimation of esds in distances, angles and torsion angles; correlations between esds in cell parameters are only used when they are defined by crystal symmetry. An approximate (isotropic) treatment of cell esds is used for estimating esds involving l.s. planes.
Refinement. All hydrogen atoms were located in difference Fourier maps and included as fixed contributions riding on attached atoms with isotropic thermal displacement parameters 1.2 or 1.5 times those of the respective carrier atom.

### Fractional atomic coordinates and isotropic or equivalent isotropic displacement parameters (Å^2^)

**Table:**

	*x*	*y*	*z*	*U*_iso_*/*U*_eq_	Occ. (<1)
Zn1	0.000000	0.000000	−0.500000	0.01420 (19)	
P1	−0.00820 (9)	0.24927 (9)	−0.750000	0.0108 (2)	
O2	−0.0548 (3)	0.3813 (3)	−0.750000	0.0223 (5)	
O1	−0.05380 (19)	0.1544 (2)	−0.61573 (16)	0.0187 (4)	
N1	0.2927 (3)	0.4198 (3)	−0.6296 (2)	0.0256 (5)	
H1A	0.395890	0.485001	−0.628272	0.031*	
H1B	0.239885	0.386350	−0.551784	0.031*	
C1	0.2161 (4)	0.3713 (4)	−0.750000	0.0148 (6)	
O3	−0.333333	0.333333	−0.8104 (8)	0.0351 (16)	0.5
H3	−0.238480	0.340450	−0.762355	0.15 (4)*	0.5

### Atomic displacement parameters (Å^2^)

**Table:**

	*U*^11^	*U*^22^	*U*^33^	*U*^12^	*U*^13^	*U*^23^
Zn1	0.0155 (2)	0.0155 (2)	0.0115 (3)	0.00776 (12)	0.000	0.000
P1	0.0106 (4)	0.0102 (4)	0.0112 (4)	0.0049 (3)	0.000	0.000
O2	0.0176 (12)	0.0148 (12)	0.0367 (14)	0.0096 (10)	0.000	0.000
O1	0.0184 (8)	0.0226 (9)	0.0144 (8)	0.0099 (7)	0.0019 (6)	0.0064 (6)
N1	0.0158 (10)	0.0259 (11)	0.0232 (11)	0.0015 (9)	−0.0035 (8)	0.0017 (8)
C1	0.0121 (14)	0.0104 (14)	0.0220 (15)	0.0057 (12)	0.000	0.000
O3	0.0222 (19)	0.0222 (19)	0.061 (4)	0.0111 (10)	0.000	0.000

### Geometric parameters (Å, º)

**Table:**

Zn1—O1^i^	2.0927 (16)	P1—O1	1.4957 (15)
Zn1—O1^ii^	2.0927 (16)	P1—C1	1.851 (3)
Zn1—O1^iii^	2.0927 (16)	N1—H1A	0.8600
Zn1—O1	2.0927 (16)	N1—H1B	0.8600
Zn1—O1^iv^	2.0927 (16)	N1—C1	1.310 (3)
Zn1—O1^v^	2.0927 (16)	O3—O3^vii^	1.147 (15)
P1—O2	1.527 (2)	O3—H3	0.9830
P1—O1^vi^	1.4957 (15)		
			
O1^i^—Zn1—O1	85.04 (6)	O2—P1—C1	101.65 (14)
O1^iv^—Zn1—O1^iii^	85.04 (6)	O1^vi^—P1—O2	112.38 (8)
O1^iv^—Zn1—O1^ii^	94.96 (6)	O1—P1—O2	112.38 (8)
O1^i^—Zn1—O1^iv^	180.0	O1^vi^—P1—O1	116.94 (14)
O1^i^—Zn1—O1^ii^	85.04 (6)	O1^vi^—P1—C1	105.92 (8)
O1—Zn1—O1^iv^	94.96 (6)	O1—P1—C1	105.92 (8)
O1—Zn1—O1^iii^	180.0	P1—O1—Zn1	140.57 (10)
O1^i^—Zn1—O1^v^	94.96 (6)	H1A—N1—H1B	120.0
O1^v^—Zn1—O1^iii^	94.96 (6)	C1—N1—H1A	120.0
O1—Zn1—O1^v^	85.04 (6)	C1—N1—H1B	120.0
O1—Zn1—O1^ii^	94.96 (6)	N1—C1—P1	119.02 (15)
O1^iv^—Zn1—O1^v^	85.04 (6)	N1^vi^—C1—P1	119.02 (15)
O1^v^—Zn1—O1^ii^	180.0	N1—C1—N1^vi^	121.6 (3)
O1^i^—Zn1—O1^iii^	94.96 (6)	O3^vii^—O3—H3	62.3
O1^iii^—Zn1—O1^ii^	85.04 (6)		
			
O2—P1—O1—Zn1	−161.51 (15)	O1—P1—C1—N1^vi^	155.6 (2)
O2—P1—C1—N1	86.8 (2)	O1^vi^—P1—C1—N1^vi^	30.8 (3)
O2—P1—C1—N1^vi^	−86.8 (2)	O1—P1—C1—N1	−30.8 (3)
O1^vi^—P1—O1—Zn1	66.3 (2)	C1—P1—O1—Zn1	−51.35 (19)
O1^vi^—P1—C1—N1	−155.6 (2)		

Symmetry codes: (i) *y*, −*x*+*y*, −*z*−1; (ii) −*x*+*y*, −*x*, *z*; (iii) −*x*, −*y*, −*z*−1; (iv) −*y*, *x*−*y*, *z*; (v) *x*−*y*, *x*, −*z*−1; (vi) *x*, *y*, −*z*−3/2; (vii) −*x*+*y*−1, −*x*, −*z*−3/2.

### Hydrogen-bond geometry (Å, º)

**Table:**

*D*—H···*A*	*D*—H	H···*A*	*D*···*A*	*D*—H···*A*
N1—H1*A*···O2^viii^	0.86	2.19	2.925 (3)	143
N1—H1*B*···O1^i^	0.86	2.16	2.926 (3)	147
O3—H3···O2	0.98	1.59	2.520 (3)	155

Symmetry codes: (i) *y*, −*x*+*y*, −*z*−1; (viii) −*y*+1, *x*−*y*+1, *z*.
